# Complete Genome Sequence of Coxsackievirus B1 Isolated during Case Outbreaks in 2007 in the United States

**DOI:** 10.1128/genomeA.00574-14

**Published:** 2014-07-24

**Authors:** Kevin K. Quinn, Susan K. Wollersheim, Paul Krogstad

**Affiliations:** a Department of Pediatrics, David Geffen School of Medicine at UCLA, Los Angeles, California, USA; b Department of Molecular and Medical Pharmacology, David Geffen School of Medicine at UCLA, Los Angeles, California, USA

## Abstract

In 2007, clusters of severe coxsackievirus B1 (CVB1) infection occurred across the United States, and CVB1 became the most commonly reported enterovirus. The complete genome sequence of CVB1 isolated from an infant (CVB1-Chi07) was examined and found to be divergent from the Conn5 reference strain, with 80% and 96% similarities at the nucleotide and amino acid levels, respectively.

## GENOME ANNOUNCEMENT

Coxsackievirus B1 (CVB1) belongs to the *Enterovirus B* species (within the genus *Enterovirus*, family *Picornaviridae*). CVB1 was first recognized in 1948 (CVB1-Conn5 strain) and then was sporadically identified as one of the top five enteroviruses circulating in the United States in 1977, 1991, and 1992 (1). In 2007, it reemerged with nationwide clusters of cases and became the most commonly isolated enterovirus in the United States (2). While CVB1 was not previously associated with myocarditis or fatalities in infants (1, 3), CVB1-Chi07 caused 6 neonatal deaths among 176 identified infections (4–6). Heart failure was a prominent clinical feature (5). An analysis of the CVB1-Chi07 virus was initiated in an effort to determine the cause of its newfound virulence and cardiotropism.

CVB1-Chi07 was originally obtained from Stanford Shulman at Northwestern University and propagated in HeLa-RW cells (7). Full-length sequencing was performed with custom oligonucleotide primers, and the raw sequence data were assembled using BioEdit version 7.2.3. Nucleic acid and protein sequence alignments were done with BioEdit version 7.2.3. Differences between the viruses were calculated with the NIH BLAST software suite. Bootscan analysis was performed using the SimPlot program (version 3.5.1).

The overall genome organization of this new CVB1 strain was similar to those of other enteroviruses. An analysis of the 5′ untranslated region containing the internal ribosome entry site revealed that the CVB1-Chi07 stem-loop 2 structure, previously noted to be associated with myocarditic potential (8), was only 64.7% identical to that of the reference CVB1-Conn5 strain, while stem-loops 1 and 3 to 7 were 85.7 to 98.8% identical, suggesting a possible etiology of the newly recognized cardiovirulence of CVB1-Chi07. Following the 5′ untranslated region (UTR), the single open reading frame (6,546 bp) encodes a polyprotein precursor of 2,182 amino acids. The CVB1-Chi07 sequence was most closely related to a CVB1 strain identified in 2011, with accession no. JN797615 (98% amino acid identity, with 92% nucleotide identity). The capsid (P1) nucleotide sequence of CVB1-Chi07 was 96% identical to JN797615 and 80% identical to the reference CVB1 M16560 (with 99% and 96% amino acid identities, respectively). The P2 gene sequences were 93% and 79% identical (with 97% and 98% amino acid identities, respectively). The P3 gene sequences were 84% and 78% identical (with 97% and 95% amino acid identities, respectively). Bootscan analysis revealed no recombination events between CVB1-Chi07 and sequences related to JN797615, the CVB1-Conn5 strain, or the reference strains of the top 15 circulating enteroviruses known to circulate in the United States between 1975 and 2008 (1–3).

### Nucleotide sequence accession number.

This sequence has been deposited in GenBank under the accession no. KJ849619.
